# Changes in T Lymphocytes and Cytokines After Anti-TNF Treatment in Pediatric Inflammatory Bowel Disease: Association with Response to Pharmacologic Therapy

**DOI:** 10.3390/ijms26073323

**Published:** 2025-04-02

**Authors:** Paula Zapata-Cobo, Sara Salvador-Martín, Sergio Gil-Manso, Marta Velasco Rodríguez-Belvís, Laura M. Palomino, Ana Moreno-Álvarez, Begoña Pérez-Moneo, Ruth García-Romero, María J. Fobelo, Diana García-Tirado, César Sánchez, Gemma Pujol-Muncunill, Oscar Segarra, Montserrat Montraveta, Lorena Magallares, Rafael Correa-Rocha, María Sanjurjo-Sáez, Marjorie Pion, Luis A. López-Fernández

**Affiliations:** 1 Servicio de Farmacia, Hospital General Universitario Gregorio Marañón, Instituto de Investigación Sanitaria Gregorio Marañón, 28007 Madrid, Spain; paula.zapata@iisgm.com (P.Z.-C.); sara.salvador@salud.madrid.org (S.S.-M.); maria.sanjurjo@salud.madrid.org (M.S.-S.); 2 Laboratory of Immuno-Regulation, Hospital General Universitario Gregorio Marañón, Instituto de Investigación Sanitaria Gregorio Marañón, 28007 Madrid, Spain; sg4528@cumc.columbia.edu (S.G.-M.); rafael.correa@iisgm.com (R.C.-R.); 3 Sección de Gastroenterología y Nutrición Pediátrica, Hospital Infantil Universitario Niño Jesús, 28007 Madrid, Spain; mvelascor@salud.madrid.org (M.V.R.-B.); lauramaria.palomino@salud.madrid.org (L.M.P.); 4 Complejo Hospitalario Universitario A Coruña, 15006 A Coruña, Spain; ana.moreno.alvarez@sergas.es; 5 Hospital Universitario Infanta Leonor, 28031 Madrid, Spain; begona.perezm@salud.madrid.org; 6 Hospital Universitario Miguel Servet, 50009 Zaragoza, Spain; rgarciarom@salud.aragon.es; 7 Hospital Universitario Virgen de Valme, Universidad de Sevilla, 41014 Sevilla, Spain; mariaj.fobelo.sspa@juntadeandalucia.es; 8 Hospital Universitario Parc Taulí, 08208 Sabadell, Spain; dgarciat@tauli.cat; 9 Sección de Gastroenterología, Hepatología y Nutrición Infantil, Hospital General Universitario Gregorio Marañón, Instituto de Investigación Sanitaria Gregorio Marañón, 28009 Madrid, Spain; cesar.sanchez.sanchez@salud.madrid.org; 10 Servicio de Gastroenterología, Hepatología y Nutrición Pediátrica, Hospital Sant Joan de Déu, 08950 Barcelona, Spain; gemma.pujol@sjd.es; 11 Hospital Vall d’Hebrón, 08035 Barcelona, Spain; oscar.segarra@vallhebron.cat; 12 Hospital Universitario Germans Trias i Pujol, 08916 Barcelona, Spain; mmontraveta.germanstrias@gencat.cat; 13 Hospital Universitario La Paz, 28046 Madrid, Spain; lorena.magallares@salud.madrid.org; 14 Advanced ImmunoRegulation Group, Instituto de Investigación Sanitaria Gregorio Marañón, 28007 Madrid, Spain

**Keywords:** infliximab, adalimumab, Treg, pediatric, response biomarkers, T-lymphocytes, cytokines, inflammatory bowel disease

## Abstract

Failure of anti-TNF therapy is a real concern in children with inflammatory bowel disease (IBD) owing to the limited therapeutic arsenal. Anti-TNF drugs modulate the immune response, a key driver of chronic inflammation in IBD. Accordingly, we analyzed changes in the frequency of T-lymphocyte and cytokine levels after 6 weeks of treatment to identify potential biomarkers of response to anti-TNF drugs. We recruited 77 patients under 18 years of age diagnosed with IBD and treated with an anti-TNF drug. Using flow cytometry and multiplex ELISA, we analyzed 31 T-lymphocyte populations and four cytokines. We identified changes in 10 populations of T lymphocytes after 6 weeks of treatment. Naïve Tregs were associated with a primary response to anti-TNF drugs, while activated Tregs were associated with long-term response. Serum INF-γ levels were decreased after anti-TNF treatment in children with Crohn’s disease (CD), but not in those with ulcerative colitis (UC). The memory CD8+ Type 2 Cytotoxic T (Tc2) subset increased in non-responders with CD and the CD4+ memory Th17 cells increased in non-responders with UC. These findings could help us to understand the cellular regulation of anti-TNF therapy, to identify children at a higher risk of treatment failure, and, potentially, to develop more personalized therapeutic strategies.

## 1. Introduction

Inflammatory bowel disease (IBD) is an immune-mediated disorder that comprises ulcerative colitis (UC), Crohn’s disease (CD), and indeterminate IBD. The etiology of these diseases is multifactorial and includes genetic, environmental, and microbiome-related factors [1]. The influence of these factors differs depending on age at diagnosis, with environment proving more relevant when the disease debuts at a younger age [2]. However, for children diagnosed before the age of 6 (very early onset IBD), genetics is the main factor [3].

The gut barrier is an essential part of the digestive system, which is normally compromised in patients with IBD. Dysregulation of the immune system contributes to chronic inflammation of the gut barrier. Multiple studies have demonstrated how different types of T lymphocyte contribute to the pathogenesis of IBD [4,5]. More specifically, the balance between T helper 17 (Th17) and regulatory T cells (Tregs) is considered essential in the pathogenesis and progression of IBD [6]. Higher levels of the pro-inflammatory cytokine IL-17, along with the increased abundance of Th17 cells, its primary source, have been observed in the inflamed tissues of IBD patients [7,8]. In contrast, Tregs have anti-inflammatory capacity by acting as suppressors of Th17 cell activity [9]. A deficiency of circulating Tregs in active IBD has been documented [10], although the frequency of functional Tregs increases in patients treated with anti-TNF drugs [10,11]. In addition, other T-lymphocyte populations, such as cytotoxic T lymphocytes, have also been considered relevant in IBD [12].

Cytokines produced by immune cells also participate in the development and perpetuation of IBD [13]. The signature cytokines for both UC and CD are driven by the IL-23 and TNF-α axes [14]. For this reason, anti-TNF drugs are widely used to treat IBD.

Despite the marked differences between pediatric-onset IBD and adult-onset IBD [15], comparatively little is known about IBD in children [16]. Moreover, the only two biological treatments approved for children, infliximab and adalimumab, bind to TNF and block its pro-inflammatory effect [17]. Nevertheless, limited data are available regarding changes in T-cell and cytokine profiles in pediatric IBD treated with both anti-TNF drugs. For this reason, it is important to understand the physiological impact of such drugs when optimizing biological therapies in children with IBD in order to achieve a better prognosis. In a very recent study, the authors observed increased frequencies of conventional memory and regulatory memory T cells in children with IBD treated with infliximab [18]. However, more complete, comprehensive T-cell profiling is necessary if we are to understand the effect of anti-TNF drugs on children with IBD.

Therefore, we analyzed 31 populations of T lymphocytes and the levels of four cytokines in peripheral blood in children with IBD treated with anti-TNF drugs, assessing samples taken before initiation of treatment and after 6 weeks of treatment. Our goal was to identify the changes in these cells and cytokine levels induced by the anti-TNF drugs and to determine potential associations with treatment response.

## 2. Results

### 2.1. Patient Characteristics

**Figure 1:**
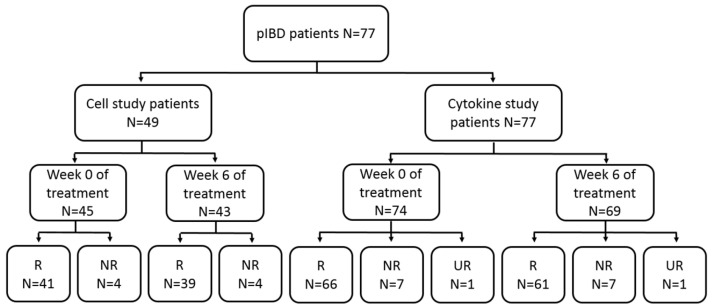
Flow chart showing the selection of study patients. The patients included in each study were divided into week 0 and week 6 and further classified into primary responders and primary non-responders. No response data were obtained for 1 patient included in the cytokine study. R, responders; NR, non-responders; UR, unclassified response.

A total of 77 patients (Figure 1) met the inclusion criteria and were included in the cytokine study (see Table 1 for patient characteristics). Evaluation of the response revealed 69 patients to be primary responders to anti-TNF drugs and 7 to be non-responders. The clinical data necessary to evaluate the response were missing in one case. Type of IBD was the only variable that differed significantly between the groups (*p* value 0.025) (Table 1). This may be because most patients had CD (80.5%); of these, 93.5% responded to treatment. The remainder (19.5%) had UC; of these, 73.3% were responders.

It was not possible to collect samples for analysis from all patients at week 0 of treatment or at week 6. Some patients had samples only at week 0, and others only at week 6. These were used for independent analyses at the respective time points. For the cytokine study, we obtained 74 samples before initiation of therapy and 69 after 6 weeks. For the cell study, 45 samples were collected at week 0 and 43 at week 6 (Figure 1).

Only 49 patients had complete data for the cell study (45 responders and 4 non-responders) (Table 2). As in the cohort of patients for the cytokine study, the type of IBD was the only characteristic for which differences between responders and non-responders were statistically significant (*p* value 0.023). Patients diagnosed with UC were overrepresented in the non-responder group (30%) compared to those with CD (2.6%), despite being less represented overall (20.4%).

### 2.2. Effects of Anti-TNF Drugs on the T-Lymphocyte Profile After 6 Weeks of Treatment

The percentages of the 31 T lymphocyte subsets (Table 3) were analyzed.

**Figure 2:**
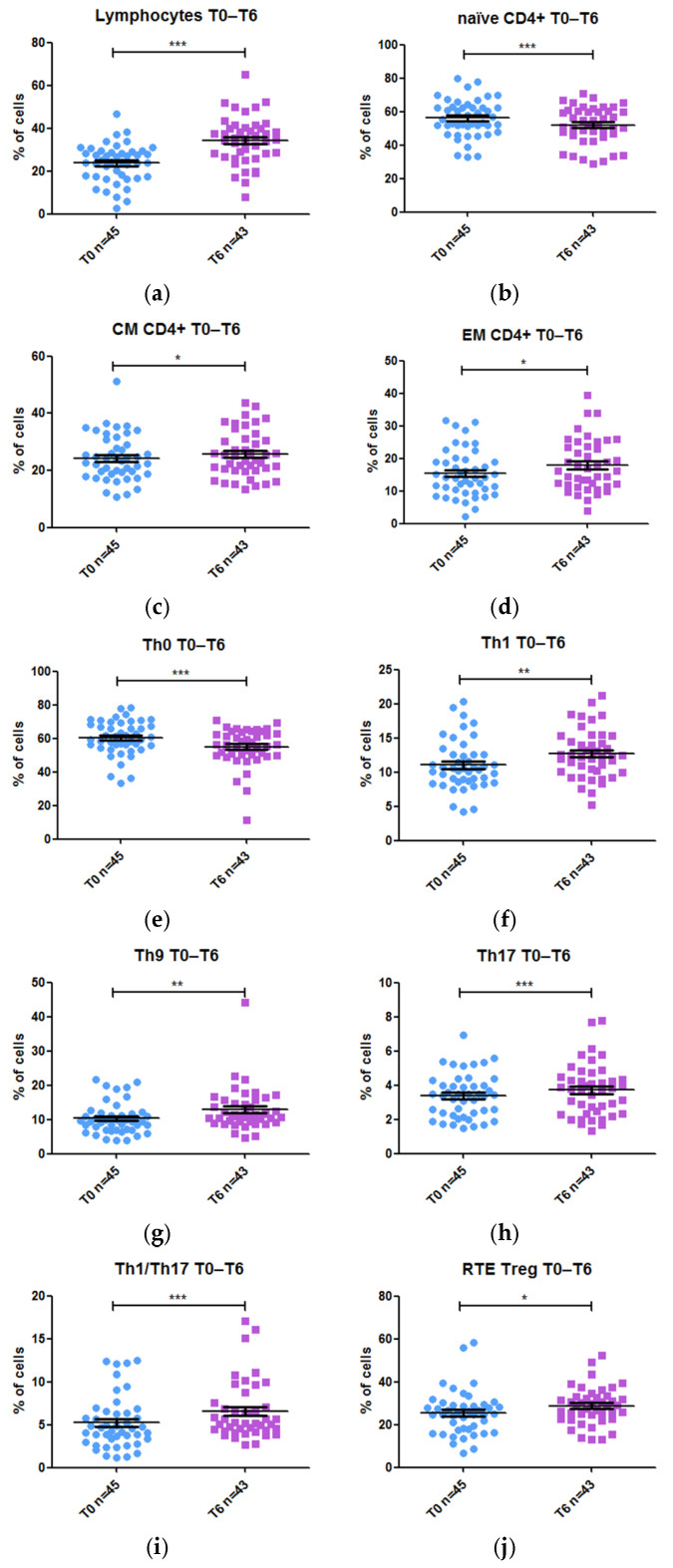
Frequency of T-cell populations before and after 6 weeks of treatment with an anti-TNF drug: (**a**) total lymphocytes (gated on leukocytes); (**b**) naïve CD4+ (gated on CD4+ T-cells); (**c**) CM CD4+ (gated on CD4+ T-cells); (**d**) EM CD4+ (gated on CD4+ T-cells); (**e**) Th0 (gated on CXCR3- CCR4- CD4+ T-cells); (**f**) Th1 (gated on CXCR3+ CCR4- CD4+ T-cells); (**g**) Th9 (gated on CD4+ T-cells); (**h**) Th17 (gated on CXCR3- CCR4+ CD4+ T-cells); (**i**) Th1/Th17 (gated on CXCR3+ CCR4- CD4+ T-cells); (**j**) RTE Treg (gated on Treg cells). * *p* value < 0.05; ** *p* value < 0.01; *** p value < 0.001.

Changes in frequency of these 31 T populations were analyzed at week 0 and at week 6 (Figure 2). After 6 weeks of anti-TNF drug treatment, a significant increase was observed in the frequency of total lymphocytes (1.46-fold, *p* value 0.000004), and also in the following T-lymphocyte subsets: Central Memory (CM) CD4 (1.08-fold, *p* value 0.037), Effector Memory (EM) CD4 (1.15-fold, *p* value 0.017), Th1 (1.16-fold, *p* value 0.002), Th9 (1.13-fold, *p* value 0.001), Th17 (1.10-fold, *p* value 0.000189), Th1/Th17 (1.16-fold, *p* value 0.000373), and recent thymic emigrants (RTE) Tregs lymphocyte (1.15-fold, *p* value 0.014). In contrast, the frequency of naïve CD4 (−0.94-fold, *p* value 0.000151) and Th0 (−0.93-fold, *p* value 0.000007) decreased after 6 weeks of biological treatment. We did not find any statistically significant difference in the frequencies of the remaining 21 T-lymphocyte subsets.

Our study of the evolution of these populations once anti-TNF treatment was complete and after separating patients by disease type revealed a series of differences. In CD, there was a significant increase in the frequency of the subset total lymphocytes (1.37-fold, *p* value <0.001), EM CD4 (1.17-fold, *p* value 0.008), Th1 (1.20-fold, *p* value 0.017), Th9 (1.20-fold, *p* value 0.002), Th17 (1.13-fold, *p* value 0.001), Th1/Th17 (1.24-fold, *p* value 0.001), and RTE Tregs (1.10-fold, *p* value 0.006). In contrast, there was a significant decrease in the percentage of naïve CD4 (−0.95-fold, *p* value 0.001) and Th0 (−0.95-fold, *p* value <0.001). In UC, the differences were only significant in the frequency of total lymphocytes, Th0 and Th1. Total lymphocytes (1.60-fold, *p* value 0.025) and Th1 (1.14-fold, *p* value 0.05) increased after 6 weeks, while Th0 decreased (−0.95-fold, *p* value 0.036).

### 2.3. T Lymphocytes as Cellular Markers of Primary Response to Anti-TNF Drugs

The frequency of T lymphocytes before and after 6 weeks of anti-TNF therapy was compared between primary responders and non-responders. None of the T-cell subsets found to have changed in frequency between baseline and 6 weeks were associated with either primary or long-term response to anti-TNF therapy.

At week 0, the percentage of Th17 (gated on CXCR3- CCR4+ CD4+ T-cells) was lower in non-responders than in responders, while the frequencies of EM CD8 (gated on CD8+ T-cells) and DP CD4+ CD8+ (gated on total lymphocytes) were higher in non-responders than in responders. None of these differences was statistically significant.

**Figure 3:**
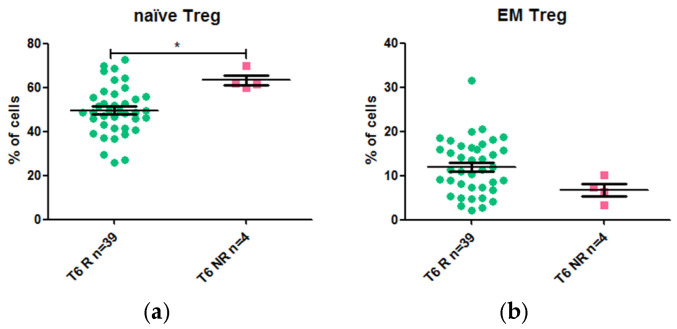
Frequency of T-cell populations in responders and non-responders after 6 weeks of treatment with an anti-TNF drug: (**a**) Naïve Tregs (gated on Treg cells); (**b**) EM Tregs (gated on Treg cells). * *p* value < 0.05.

After 6 weeks of biological therapy, only the frequency of naïve Tregs was associated with response to anti-TNF drugs (Figure 3). The median percentage of naïve Tregs (gated on Treg cells) was 61.7% in non-responders compared to 48.8% in responders. EM Tregs (gated on Treg cells) decreased in non-responders, although the difference was not statistically significant.

### 2.4. T Lymphocytes as Cellular Markers of Long-Term Response

**Figure 4:**
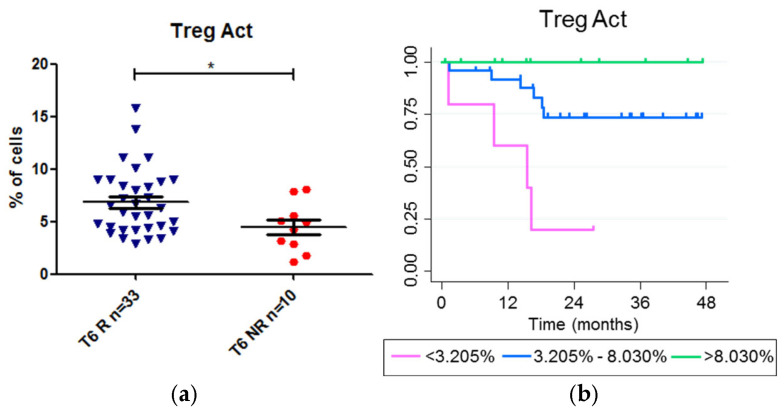
A low frequency of Act Treg cells (gated on Treg cells) after 6 weeks of anti-TNF treatment is associated with long-term failure of anti-TNF drugs. (**a**) Comparison of the frequency of Act Treg in patients whose anti-TNF therapy failed (NR) or did not fail (R) during the follow-up; (**b**) Kaplan–Meier curve of patients with high (>8.030%), medium (3.205% to 8.030%), and low (<3.205%) frequency of Act Treg cells. * *p* value < 0.05.

We also analyzed whether we could predict the long-term response before starting treatment or after 6 weeks. For this purpose, we first compared the frequency of the T-lymphocyte populations at 0 and 6 weeks in patients whose anti-TNF drug failed at any time during the follow-up and in patients whose therapy did not fail. The only statistically significant difference was observed at week 6. The median percentage of Act Tregs (gated on Treg cells) was lower in the patients whose therapy failed (non-responders) (4.64) than in those whose therapy did not fail (R) (6.25) (*p* value 0.036), suggesting that in patients with a lower frequency of Act Treg cells, anti-TNF therapy failed earlier (Figure 4a)

We performed a Kaplan–Meier analysis to further investigate this hypothesis. Three groups were established based on the frequency of Act Treg lymphocytes (gated on total Tregs) at 6 weeks after treatment, and Receiver Operating Characteristic (ROC) curves were used to obtain three cut-off points: low-Act Treg frequency, <3.205%; medium-Act Treg frequency, between 3.205% and 8.030%; and high-Act Treg frequency, >8.030%. The results showed that patients with a lower percentage of Act Treg at week 6 tended to experience failure earlier than those with a higher percentage (*p* value = 0.003) (Figure 4b).

### 2.5. Cytokine Profile Before and After 6 Weeks of Biological Therapy

**Figure 5:**
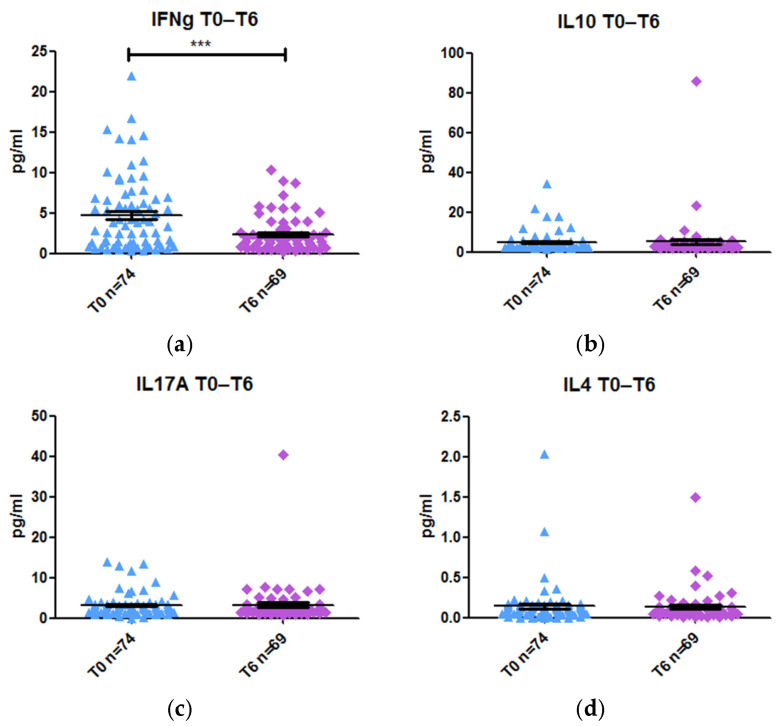
Levels of cytokines prior (T0) and after 6 weeks of treatment (T6) with anti-TNF therapy: (**a**) IFN-γ; (**b**) IL-10; (**c**) IL-17A; (**d**) IL-4. *** *p* value < 0.001.

We compared the levels of IFN-γ, IL-10, IL-17A, and IL-4 at week 0 and week 6 of treatment in peripheral blood. Only IFN-γ levels decreased significantly after anti-TNF therapy (−2.09-fold, *p* value < 0.0001), from a concentration of 3.41 pg/mL to 1.63 pg/mL (Figure 5a). IL-10, IL-17A, and IL-4 levels remained unchanged after treatment (Figure 5b–d).

**Figure 6:**
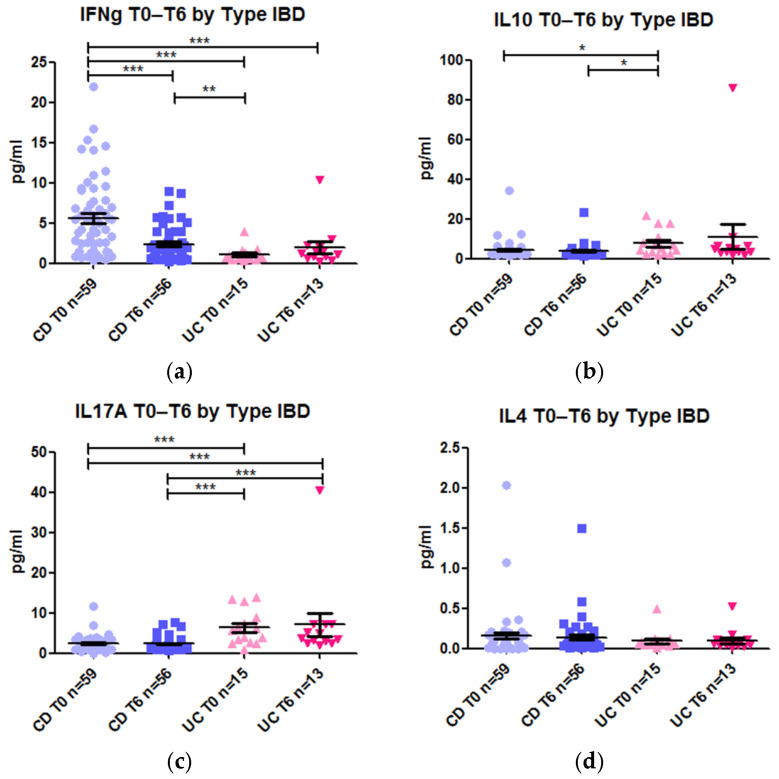
Levels of cytokines stratified by time of administration of the anti-TNF agent, namely, week 0 (T0) or week 6 (T6), and type of disease, namely, CD or UC. (**a**) IFN-γ; (**b**) IL-10; (**c**) IL-17A; (**d**) IL-4. * *p* value < 0.05; ** *p* value < 0.01; *** *p* value < 0.001.

We also compared cytokine levels between CD and UC patients at weeks 0 and 6. There was a significant reduction in IFN-γ levels in CD (−2.94-fold, *p* value <0.0001) from 4.99 pg/mL to 1.70 pg/mL after 6 weeks of treatment (Figure 6a), but not in UC. Moreover, prior to treatment, IFN-γ levels were higher in CD than in UC (4.99 pg/mL vs. 0.70 pg/mL; *p* value <0.0001) (Figure 6a). In contrast, IL-17A levels in plasma were significantly higher in UC than in CD (5.59 pg/mL vs. 1.96 pg/mL; *p* value <0.0001). This difference remained unchanged after 6 weeks of therapy (*p* value 0.0001) (Figure 6c). IL-10 levels were also higher in UC patients before treatment (*p* value 0.034) (Figure 6b). Finally, IL-4 levels did not differ significantly between the subgroups (Figure 6d).

In summary, UC and CD displayed different cytokine profiles, with CD showing higher IFN-γ levels and UC showing higher IL-10 and IL-17A levels. However, anti-TNF treatment did not induce significant changes in plasma cytokine levels. The only notable change was a significant reduction in IFN-γ levels after 6 weeks of treatment in CD patients.

### 2.6. Cytokines as Cellular Markers of Response to Anti-TNF Drugs in Pediatric IBD

No differences in cytokine levels were observed between primary responders and non-responders at 0 and 6 weeks after the start of anti-TNF therapy. None of the four cytokines was associated with response to treatment either before or after 6 weeks of treatment. However, a trend toward a higher level for IFN-γ was observed in responders before starting treatment (3.65 vs. 0.7 pg/mL) (Table 4). Taking into account the difference in the cytokine profile depending on the type of disease, the same analysis of the response was performed separately in CD and UC. No significant results were found.

We also analyzed whether we could predict long-term response before starting treatment or after 6 weeks. For this purpose, we first compared cytokine levels at 0 and 6 weeks of anti-TNF treatment in patients whose therapy failed at any time during follow-up and in patients whose therapy did not fail. None of the cytokines analyzed was associated with response during the follow-up period. Stratification by disease type was also performed, although it did not reveal significant changes.

### 2.7. Unsupervised Analysis of T-Cell Flow Cytometry Panel

**Figure 7:**
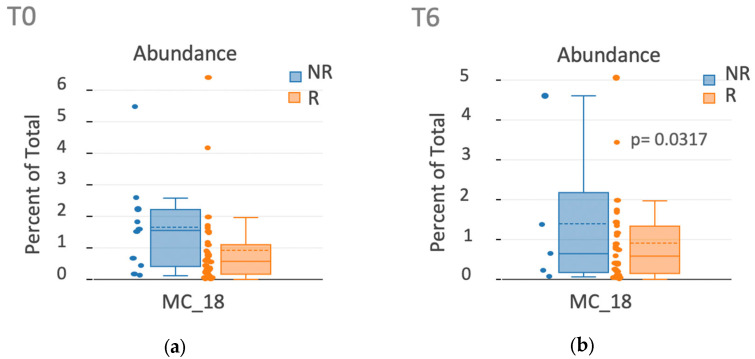

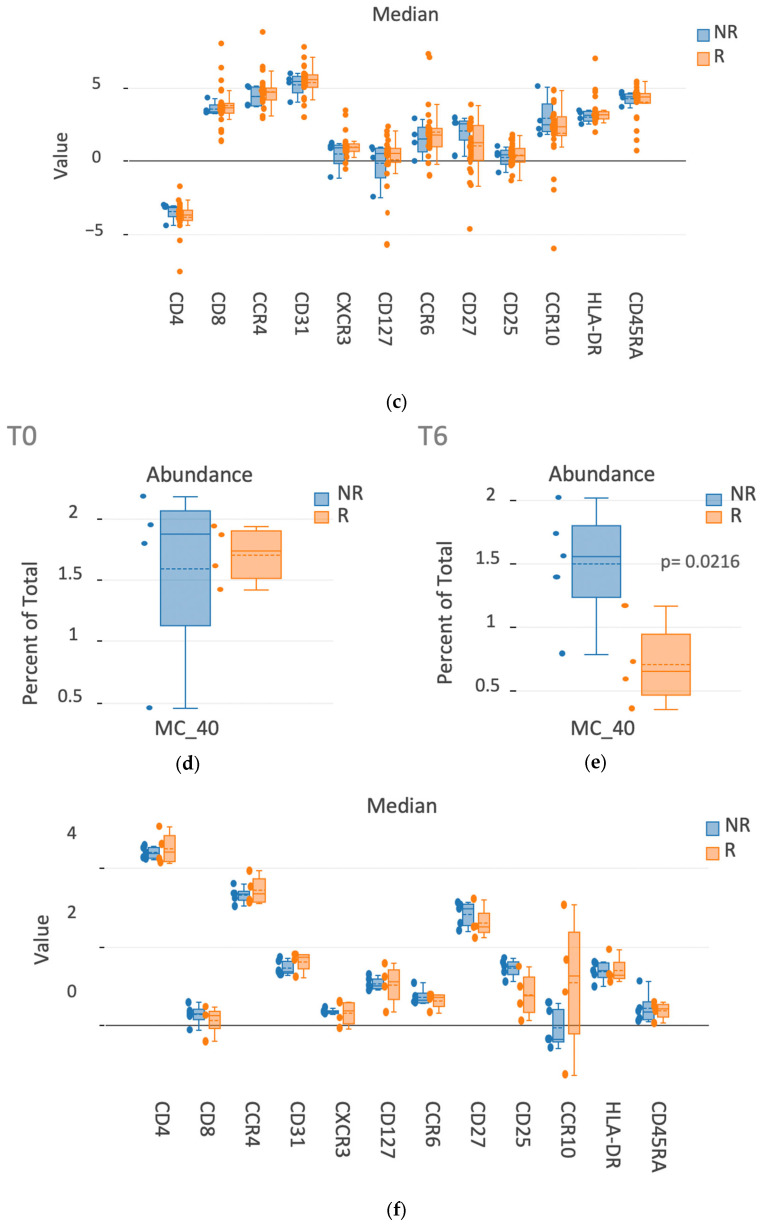
Use of unsupervised algorithms to detect non-conventional cellular subsets. (**a**) Box plot histogram showing the abundance of metacluster_18 in responders (R) and non-responders (NR) among individuals with CD at T0 and (**b**) T6 obtained through opt-SNE and FlowSOM analysis. (**c**) Arbitrary unit of the medians of fluorescence (MFI) of cellular markers in the metacluster_18. (**d**) Box plot histogram showing the abundance of metacluster_40 in responders and non-responders among individuals with UC at T0 and (**e**) T6 obtained through opt-SNE and FlowSOM analysis. (**f**) Arbitrary unit of the MFI of the cellular markers of metacluster_40. Intermediate dotted line, mean; intermediate unbroken line, median; the upper and lower boxes represent the first and third quartiles, respectively. P corresponded to the adjusted *p* value.

We applied a high-dimensional flow cytometry analysis to compare lymphocyte activation and differentiation between cohorts. Using the unsupervised algorithms (opt-SNE), we detected very few variations in the distribution of cellular populations between the UC and CD, at T0 or T6, in responders and non-responders. Using the opt-SNE results, we ran FlowSOM, through which clustering makes it possible to see how all markers are behaving in all cells. Of the 40 metaclusters generated, 1 revealed a significant difference in abundance between the responders/non-responders corresponding to a long-term failure of anti-TNF drugs at T6, but not at T0, in CD (metacluster 18, Figure 7a,b), with abundance being greater in the non-responders than in the responders. The phenotype of this metacluster was CD8+ CCR4+ CD31+ CD27 negative (neg) CD45RA intermediate (int). This subset could correspond to memory Tc2 CD8 T cells (Figure 7c). Moreover, of the 40 metaclusters generated, 1 yielded a significant difference in abundance between responders and non-responders corresponding to a long-term failure of anti-TNF drugs at T6, but not at T0, in UC individuals (metacluster 40, Figure 7d,e), with abundance being greater in the non-responders than in the responders. The phenotype of this metacluster was CD4+ CCR4+ CD27+ CD45RAneg CXCR3neg and CCR6neg, potentially corresponding to a CD4+ Th17 central memory subset (Figure 7f).

In summary, the unsupervised analyses revealed that non-responders with CD exhibited a higher abundance of CD8+ memory Tc2 subsets, while non-responders with UC showed a higher abundance of CD4+ memory Th17 cells. This sustained frequency of both subsets in non-responders is likely due to continued activation.

## 3. Discussion

The addition of biological drugs to the therapeutic arsenal of IBD has transformed how patients are managed. Biologics are used sooner after a diagnosis of IBD in children than in adults [19]. Since children must live longer with the illness, it is relevant to know the effects of these drugs on this population, especially with respect to the chronic inflammation affecting the immune system, which can lead to immune senescence. In this work, we found that the frequency of total T lymphocytes and that of nine T-lymphocyte subsets changed after 6 weeks of treatment with infliximab or adalimumab in children with IBD. In addition, we found a low frequency of naïve Tregs to be a biomarker of better primary response to anti-TNF drugs and that the frequency of activated Tregs to be a biomarker of long-term response. Finally, we observed a significant decrease in IFN-γ serum levels after 6 weeks of treatment with anti-TNF drugs in CD patients, but not in UC patients.

Surprisingly, seven populations of T lymphocytes, namely, CM CD4, EM CD4, Th1, Th17, Th9, RTE Tregs, and Th1/Th17, which are associated with differentiated and potentially pro-inflammatory functions, were more frequent after 6 weeks of treatment with infliximab or adalimumab in children with IBD. On the other hand, the frequency of naïve CD4 and Th0, which are undifferentiated subsets, decreased. Few works have studied changes in the frequencies of T lymphocytes in IBD, especially in children, and the findings are often contradictory. For instance, Bosè et al. reported no changes in the percentages of T- and B-cell lineage markers before or after anti-TNF therapy in IBD patients [20]. However, consistent with our results, an expansion of conventional memory and regulatory memory T cells has been reported in infliximab-treated children with IBD [18]. Initially, it was surprising to observe an increase in pro-inflammatory T cells after treatment with anti-TNF drugs, which have anti-inflammatory properties. However, a dual role has been reported for anti-TNF therapy, which enhances T-cell activation in peripheral blood and inhibits inflammation in target tissues [20]. Moreover, in addition to the potential pro-inflammatory properties of anti-TNF agents, it cannot be excluded that the redistribution of differentiated cells from inflamed tissue to the periphery may account for the increase in the subsets following treatment with these drugs. Thanks to the unsupervised analysis, we detected a significantly increased frequency of a cellular subset (CD8+ CCR4+ CD31+ CD27neg CD45RAint) in non-responders with CD after 6 weeks of treatment, which may be related to the memory CD8+ Tc2 subset. This subset has previously been observed in the mucosa of children with colitis, where CD8+ T cells can differentiate into non-classic Tc2 cells under stimulation with TGF-β in combination with IL-1β/TNF-α or IL-6, thus contributing to the disease [21]. The persistence of this subset after 6 weeks of treatment in non-responders with CD could be attributed to their lack of response to anti-TNF therapy, as TNF-α is one of the cytokines that contribute to their differentiation.

Our hypothesis for this study was that the T lymphocytes and/or cytokines involved in the etiology and pathogenesis of IBD could also be relevant in the response to anti-TNF drugs. In this sense, we showed that non-differentiated naïve Tregs were more frequent in non-responders than in responders after 6 weeks of treatment. Concordantly, the differentiated EM Tregs were decreased in non-responders. We also showed that higher frequencies of activated Tregs were associated with a longer relapse-free period and with response to treatment. The higher frequency of naïve Tregs and the lower frequency of EM Tregs in non-responders support the hypothesis that Tregs must undergo activation and subsequent differentiation to effectively limit inflammation. Previous studies have demonstrated that naïve Tregs lack suppressive activity until they are activated. Investigating whether the naïve Tregs in non-responders lack differentiation and activation capacity could provide insight into their inability to suppress inflammation in treated patients. The frequency of circulating and intestinal Tregs has already been observed to be higher in IBD patients than in healthy controls [22], with a notably higher Treg frequency in the intestinal mucosa than in peripheral blood. The advantages of the increased presence of Tregs in tissue are that they can reduce local inflammation and have shown healing potential, including regenerative abilities, in preclinical models [23,24,25,26]. Previously, Tregs were not identified as a marker of response to anti-TNFα treatment in CD or UC [16]. However, the authors focused on the total Treg population rather than specifically on the activated Treg subset.

To be active, Tregs must be activated, and their principal role is to mitigate inflammation induced by the immune system, particularly in hyperinflammatory conditions [27]. Our results show that the presence of peripheral activated Tregs—potentially reflecting the bowel tissue microenvironment—was strongly associated with a longer relapse-free period. Consequently, we hypothesized that, in addition to being an excellent biomarker of disease progression, activated Tregs may play an important role in the outcome of both UC and CD. However, it would be valuable to further investigate Treg functionality and phenotype, since circulating and bowel Tregs have different phenotypes [22]. In IBD patients, enrichment of Tregs with non-classical phenotypes, such as IL-17+ Tregs, which are considered pro-inflammatory and may lack the functional capacity to suppress local inflammation, has been reported [28]. The functionality of these cells is controversial and a focus of ongoing research, as deregulation of Treg biomarkers could have significant consequences in IBD and may be associated with early-onset disease [29]. For example, the absolute number of FOXP3+ IL-17A+ CD4+ T cells has been reported to be a predictor of clinical relapse in CD [30]. In other studies, IL-17+ Tregs were shown to retain suppressive function [31,32]. Therefore, it would be of great interest to perform a comprehensive characterization of their phenotype and to investigate whether these activated Tregs can migrate to and function at hyperinflammatory sites.

We also studied the effect of the anti-TNF drugs on four cytokines. In our cohort of children with IBD, INF-γ was significantly decreased (2.9-fold) after 6 weeks of treatment with anti-TNF therapy. This decrease was observed predominantly in children with CD. INF-γ is produced primarily by Th1 cells, which were increased in our study after 6 weeks of anti-TNF treatment and were strongly associated with the pathogenesis of CD [33]. This surprising inverse presence of Th1 cells and the plasma level of IFN-γ could reflect a strong decrease in the capacity of Th1 cells for secreting IFN-γ in children treated with anti-TNF drugs. Moreover, it was demonstrated that T-cell activation and proliferation were inhibited after incubation with infliximab in in vitro experiments, with a decrease in pro-inflammatory cytokines such as IFN-γ, IL-13, IL-17A, and TNF [34]. We confirmed this finding, demonstrating reduced IFN-γ production in plasma. The increased presence of the Th1 subset may result from the redistribution of this subset from inflamed tissues to the periphery following the improvement in local inflammation due to treatment.

Interestingly, plasma levels of IL-17A were significantly higher in UC patients than in CD patients. This finding supports the hypothesis that CD is more strongly driven by IFN-γ, while UC is predominantly associated with IL-17A, consistent with previous observations in treatment-naïve, newly diagnosed CD and UC patients, among whom higher levels of IL-17A were reported in UC [35,36,37]. Surprisingly, IL-17 levels did not decrease after anti-TNF therapy, as reported elsewhere [38]. Thanks to the unsupervised analysis, we also observed that the frequency of the cellular subset related to central memory Th17 CD4+ T cells remained significantly elevated in non-responders with UC after 6 weeks of treatment. This subset could be a potential source of the IL-17A detected even after treatment, particularly in individuals who experienced treatment failure. However, IL-17 is not exclusively produced by the Th17 subset; it can also be produced by Th17-like Tregs [39], which are memory Tregs that play a key role in ensuring a favorable response to treatment in this study. The potential role of Act Treg IL-17+ cells in IL-17A production remains unclear, and a deeper analysis of Treg phenotype in affected patients could provide insight into the source of IL-17.

IL-10 plays a crucial role in susceptibility to IBD, as evidenced by numerous genetic studies [40,41]. IL-10 levels were higher in patients with UC than in those with CD. These findings contrast with those of previous studies in both adults [42] and children [43]. However, consistent with other studies, treatment with anti-TNF drugs in our cohort did not significantly impact IL-10 [44].

Our study has several limitations. Firstly, comparing specific T-lymphocyte populations across studies can be challenging because of the variability between the markers used by different research groups. For example, we did not analyze the specific marker used by Schnell et al. to identify exhausted Treg populations [18]. Furthermore, the evolving definitions and identification of markers and cell populations over time can restrict direct comparison of newer studies with older ones. Given that a significant limitation arises from the improved efficacy of biological therapies in children with IBD, treatment failures are less frequent than in previous years, making it more challenging to recruit a sufficient number of primary non-responders and thus ensure a robust analysis.

Despite these limitations, this study’s findings hold significant importance, as they illuminate critical cellular mechanisms underlying anti-TNF therapy in pediatric IBD. Unlike previous research, we identified specific T-lymphocyte subsets, notably naïve and activated Tregs, as potential biomarkers for primary and long-term treatment response, respectively. Furthermore, the differential impact of anti-TNF treatment on INF-γ levels in Crohn’s disease versus ulcerative colitis, and the unique increase in memory CD8+ Tc2 cells in non-responders with CD, distinguishes our work. These results pave the way for a deeper understanding of treatment failure risks and open avenues for developing personalized therapeutic strategies, ultimately improving outcomes for young IBD patients.

## 4. Materials and Methods

### 4.1. Study Design and Patient Characteristics

We performed an observational, ambispective, longitudinal, and multicenter study. Participants were informed about the study and recruited between May 2020 and September 2022 in the pediatric departments of Spanish hospitals. The inclusion criteria were age under 18 years, diagnosis of IBD, and initiation of anti-TNF therapy (infliximab or adalimumab). Participants were followed up until July 2024.

Clinical and demographic variables were collected, as follows: sex, age at diagnosis and at initiation of treatment, type of IBD, type of anti-TNF drug, and concomitant immunomodulator. We recorded the Pediatric Crohn’s Disease Activity Index (PCDAI) and the Pediatric Ulcerative Colitis Activity Index (PUCAI), as well as C-reactive protein and fecal calprotectin at the start of treatment and during treatment.

Type of anti-TNF drug and type of IBD were analyzed as potential sources of bias. The sample size was calculated as per Salvador-Martín et al. [45].

Primary response was defined as a decrease of at least 15 points in the PCDAI/PUCAI after 14 weeks of treatment (infliximab) or after 26 weeks (adalimumab). When the initial activity index was <15, a decrease to 0 was considered a response, as was an improvement in acute phase reactant and calprotectin levels. Data were collected using an electronic data capture tool (REDCap).

### 4.2. Samples and Cell Surface Marker Staining

Blood samples were obtained by venipuncture from 49 anti-TNF therapy–naïve pediatric IBD patients before initiation and after 6 weeks of treatment. Blood was preserved in EDTA tubes and kept at 4 °C until processing by flow cytometry for the analysis of 31 T-cell populations (Table 3). We used a broad panel of T-cells designed by the Laboratory of Immunoregulation for the study of the immune system in multiple diseases.

All samples were processed within 24 h of collection in a horizontal laminar flow hood to ensure protection from external and cross-contamination. Cell surface staining was performed by incubation of 100 µL of blood for 30 min with 32 µL of antibody mix comprising the following fluorescent antibodies: CD4 (VioBlue), CD3 (VioGreen/PO), CD8a (BV570), CCR4 (BV605), CD31 (BV650), CXCR3 (VioBright FITC), CD127 (PE), CCR6 (PE-Vio615), CD27 (PerCP-Vio700/PC5. 5), CD25 (PE-Vio770/PC7), CCR10 (APC), HLA-DR (Alexa Fluor 700/APC5), and CD45RA (APC-Vio770).

After surface labeling, red blood cells were lysed using BD FACS^TM^ Lysing Solution 10× Concentrate (ref. 349202, Fisher Scientific, Waltham, MA, USA) in a 1× concentration for 15 min under darkness. Surface markers were analyzed using flow cytometry in a MACSQuant^®^ Analyzer 16 instrument (Miltenyi Biotec, Bergisch Gladbach, Germany), and cell proportion values were determined with conventional gating using Kaluza Analysis v2.1 (Beckman Coulter, Brea, CA, USA).

### 4.3. Detection of Cytokine Levels in Plasma

Cytokine levels were measured in 74 plasma samples before initiation and after 6 weeks of treatment using the microfluidic ELISA device ELLA-Protein Simple (Biotechne, Minneapolis, MN, USA). We quantified IFN-γ, IL-10, IL-17A, and IL-4 using Simple Plex Runner v. 3.7.2.0 software (Biotechne, Minneapolis, MN, USA). Measurements below the lower limit of quantification (LLOQ) or above the upper limit of quantification (ULOQ) were omitted. The LLOQ and ULOQ were 1.04–4040 pg/mL for IL-4, 2.1–20,000 pg/mL for IL17A, 0.72–2892 pg/mL for IL-10, and 0.34–8000 pg/mL for IFNg, respectively.

### 4.4. Unsupervised Analysis of the Flow Cytometry Panel

In addition to traditional manual gating based on cytometry data, we performed a high-dimensional flow cytometry analysis using the opt-SNE (optimized t-distributed stochastic neighbor embedding) algorithm in OMIQ (www.cytobank.org). opt-SNE was selected due to its ability to effectively reduce high-parameter data down to 2 dimensions while preserving local structures and enabling the visualization of subtle patterns across large datasets, which is particularly useful for the analysis of complex immune cell subsets. The analysis was based on 3,000,000 events, assessed using proportional sampling between the individuals from total events to ensure balanced representation across individuals and experimental conditions. For this panel, the unsupervised analyses were performed on CD3+ T cells, which were analyzed using a flow cytometry panel containing markers CD45, CD3, CD4, CD8, CD45RA, HLADR, CD25, CD127, CD27, CD31, CXCR3, CCR4, CCR6, and CCR10. The parameters were as follows: iteration, 5000; perplexity, 30; theta, 0.5 with a random seed for reproducibility. We then applied FlowSOM clustering algorithm (self-organizing map from flow cytometry) to the opt-SNE reduced dimension. FlowSOM uses a self-organizing map to organize cells into clusters based on their phenotypic similarities. These clusters were then grouped into higher-order metaclusters, providing insight into distinct functional immune cell subsets. The clustering method parameters were as follows: xdim, 12; ydim, 12; number of training iterations, 10; and number of metaclusters, 40, with consensus metaclustering to ensure stable and reproducible results. The use of a random seed ensured that results were consistent across runs, increasing the reliability of the clustering process.

### 4.5. Statistical Analysis

The statistical analysis was performed using IBM SPSS Statistics v.26 (IBM Corp., Armonk, NY, USA). Quantitative continuous variables were expressed as median and interquartile range (IQR), whereas qualitative variables were expressed as frequency and percentage. The Mann–Whitney test and chi-squared test (or Fisher exact test, as appropriate) were used to compare quantitative and qualitative variables, respectively. The normality of the distribution was evaluated using the Kolmogorov–Smirnov test or Shapiro–Wilk test (when *n* < 30).

Cell subtype frequencies were presented as median and interquartile range (IQR). Differences in absolute percentages of cell populations between responders and non-responders were tested using the Mann–Whitney test, as were differences in cytokine expression levels between responders and non-responders. For all tests, a *p* value < 0.05 was considered statistically significant. Graphs were plotted using GraphPad Prism v5.1 (Dotmatics, Boston, MA, USA).

### 4.6. Ethical Considerations

The study was approved by the ethics committees of the participating hospitals and conducted in accordance with the World Medical Association Declaration of Helsinki and Spanish legislation. The samples were obtained from a registered collection (c.0003459; Instituto de Salud Carlos III). Written informed consent to perform this research was obtained from the patients and/or their legal guardians. Participants aged >12 years signed an assent form.

## 5. Conclusions

We found that the frequency of total T lymphocytes, as well as that of nine specific populations of T lymphocytes, changed after 6 weeks of treatment with infliximab or adalimumab in children with IBD (CM CD4, EM CD4, Th1, Th17, Th9, RTE Tregs, and Th1/Th17 increased and naïve CD4 and Th0 decreased). In addition, the frequency of naïve Tregs after 6 weeks of anti-TNF drug treatment is a biomarker of primary response to anti-TNF drugs, while the frequency of activated and EM Tregs is a biomarker of long-term response. Finally, we observed a significant decrease in serum IFN-γ levels after 6 weeks of treatment with anti-TNF drugs in CD patients, but not in UC patients.

## Figures and Tables

**Table 1:** Demographic and clinical variables of patients for whom cytokine data were available.

Characteristics	Overall (*n* = 77)	Responders (*n* = 69)	Non-Responders (*n* = 7)	*p* Value
Sex				
Male, *n* (%)	40 (51.9%)	37 (92.5%)	2 (5%)	0.256
Female, *n* (%)	37 (48.1%)	32 (86.5%)	5 (13.5%)	
Age (years)				
At diagnosis, median (IQR, range)	12.26 (4.48, 0.92–17.26)	12.27 (4.60, 0.92–17.26)	11.91 (4.98, 6.36–16.17)	0.857
At start of treatment, median (IQR, range)	12.70 (4.74, 1.35–17.42)	12.84 (4.75, 1.36–17.42)	12.52 (5.99, 8.33–16.28)	0.921
Months from diagnosis to initiation of therapy, median (IQR, range)	2.30 (6.99, 0–63.04)	2.10 (6.70, 0–63.04)	7.36 (23.55, 0.13–27.99)	0.319
Type of IBD				
CD, *n* (%)	62 (80.5%)	58 (93.5%)	3 (4.8%)	**0.025**
IFX, *n* (%)	37 (59.7%)	37 (100%)	0	
ADL, *n *(%)	25 (40.3%)	21 (84%)	3 (12%)	
UC, *n* (%)	15 (19.5%)	11 (73.3%)	4 (26.7%)	
IFX, *n* (%)	13 (86.7%)	10 (76.9%)	3 (23.1%)	
ADL, *n *(%)	2 (13.3%)	1	1	
Anti-TNF drug				
IFX, *n* (%)	50 (64.9%)	47 (94%)	3 (6%)	0.222
ADL, *n* (%)	27 (35.1%)	22 (81.5%)	4 (14.8%)	
PCDAI at start of treatment, median (IQR, range) (*n* = 62)	27.50 (28.13, 0–175)	(*n* = 58) 28.75 (33.13, 0–175)	(*n* = 3) 5 (na, 5–20)	0.102
PUCAI at start of treatment, median (IQR, range) (*n* = 15)	55 (30, 5–85)	(*n* = 11) 50 (30, 5–70)	(*n* = 4) 65 (65, 5–85)	0.358
CRP at start of treatment (mg/L), median (IQR, range)	11 (35.38, 0.10–153.20)	12.10 (35.25, 0.10–140)	5 (86.92, 0.60–125)	0.634
FC at start of treatment (µg/g), median (IQR, range)	2000 (2220, 148–10,000)	2000 (2151, 148–10,000)	1235 (2378, 715–7595)	0.760
Type of immunotherapy				
Azathioprine, *n* (%)	58 (75.3%)	51 (87.9%)	6 (10.3%)	1
Mercaptopurine, *n* (%)	0	0	0	
Methotrexate, *n* (%)	1 (1.3%)	1 (100%)	0	
None, *n* (%)	18 (23.4%)	17 (94.4%)	1 (5.6%)	

Bold, *p* value < 0.05; ADL, adalimumab; CD, Crohn’s disease; CRP, C-reactive protein; FC, fecal calprotectin; IBD, inflammatory bowel disease; IFX, infliximab; IQR, interquartile range; PCDAI, Pediatric Crohn’s Disease Activity Index; PUCAI, Pediatric Ulcerative Colitis Activity Index; UC, ulcerative colitis. na, not applicable.

**Table 2:** Demographic and clinical variables of patients for whom cell data were available.

Characteristics	Overall (*n* = 49)	Responders (*n* = 45)	Non-Responders (*n* = 4)	*p* Value
Sex				
Male, *n* (%)	24 (49%)	23 (95.8%)	1 (4.2%)	0.609
Female, *n* (%)	25 (51%)	22 (88%)	3 (12%)	
Age (years)				
At diagnosis, median (IQR, range)	12.21 (4.66, 0.92–17.26)	12.27 (4.66, 0.92–17.26)	10.99 (6.96, 6.36–14.97)	0.622
At start of treatment, median (IQR, range)	12.84 (4.88, 1.36–17.27)	12.85 (4.71, 1.36–17.27)	11.09 (6.41, 8.33–16.28)	0.609
Months from diagnosis to initiation of therapy, median (IQR, range)	3.35 (8.28, 0.0–63.04)	3.35 (7.29, 0–63.04)	9.08 (20.98, 0.13–23.69)	0.476
Type of IBD				
CD, *n* (%)	39 (79.60%)	38 (97.4%)	1 (2.6%)	**0.023**
IFX, *n* (%)	27 (69.2%)	27 (100%)	0	
ADL, *n *(%)	12 (30.8%)	11 (91.7%)	1 (8.3%)	
UC, *n* (%)	10 (20.4%)	7 (70%)	3 (30%)	
IFX, *n* (%)	8 (80%)	6 (75%)	2 (25%)	
ADL, *n *(%)	2 (20%)	1 (50%)	1 (50%)	
Anti-TNF drug				
IFX, *n* (%)	35 (71.4%)	33 (94.3%)	2 (5.7%)	0.568
ADL, *n* (%)	14 (28.6%)	12 (85.7%)	2 (14.3%)	
PCDAI at start of treatment, median (IQR, range) (*n* = 39)	30 (32.5, 0–175)	*n* = 38 31.25 (33.13, 0–175)	*n* = 1 20 (0, 0–20)	0.624
PUCAI at start of treatment, median (IQR, range) (*n* = 10)	52.50 (52.50, 5–75)	*n* = 7 50 (50, 5–65)	*n* = 3 55 (na, 5–75)	0.730
CRP at start of treatment (mg/L), median (IQR, range)	14.20 (30.60, 0.10–130.30)	14.2 (34.95, 0.10–130.30)	8 (67.22, 0.60–88.22)	0.535
FC at start of treatment (µg/g), median (IQR, range)	2000 (1996.5, 148–7595)	1685 (1868.5, 148–6000)	2930 (5393, 715–7595)	0.182
Type of immunotherapy				
Azathioprine, *n* (%)	40 (81.6%)	36 (90%)	4 (10%)	1
Mercaptopurine, *n* (%)	0	0	0	
Methotrexate, *n* (%)	0	0	0	
None, *n* (%)	9 (18.4%)	9 (100%)	0	

Bold, *p* value < 0.05; ADL, adalimumab; CD, Crohn’s disease; CRP, C-reactive protein; FC, fecal calprotectin; IBD, inflammatory bowel disease; IFX, infliximab; IQR, interquartile range; PCDAI, Pediatric Crohn’s Disease Activity Index; PUCAI, Pediatric Ulcerative Colitis Activity Index; UC, ulcerative colitis. na, not applicable.

**Table 3:** T-cell populations studied and cell surface markers detected.

T Cell Population	Cell Surface Markers
Lymphocytes	FSC/SSC CD45+
CD3	CD3+
CD4	CD3+ CD4+
naïve CD4	CD3+ CD4+ CD45RA+ CD27+
CM CD4 (Central memory)	CD3+ CD4+ CD45RA- CD27+
EM CD4 (Effector memory)	CD3+ CD4+ CD45RA- CD27-
TemRA CD4 (Terminal effector memory)	CD3+ CD4+ CD45RA+ CD27-
CD4 Act (Activated)	CD3+ CD4+ CD45RA- HLADR+
CD4 RTE (Recent thymic emigrants)	CD3+ CD4+ CD45RA+ CD31+
Th0	CD3+ CD4+ CXCR3- CCR4- CCR6- CCR10-
Th1	CD3+ CD4+ CXCR3+ CCR4- CCR6- CCR10-
Th2	CD3+ CD4+ CXCR3- CCR4+ CCR6- CCR10-
Th9	CD3+ CD4+ CCR4- CCR6+
Th17	CD3+ CD4+ CXCR3- CCR4+ CCR6+ CCR10-
Th1/Th17	CD3+ CD4+ CXCR3+ CCR4- CCR6+ CCR10-
Th22	CD3+ CD4+ CCR4+ CCR6+ CXCR3- CCR10+
Treg	CD3+ CD4+ CD25+ CD27low
naïve Treg	CD3+ CD4+ CD25+ CD127low CD45RA+ CD27+
CM Treg (Central memory)	CD3+ CD4+ CD25+ CD127low CD45RA- CD27+
EM Treg (Effector memory)	CD3+ CD4+ CD25+ CD127low CD45RA- CD27-
TemRA Treg (Terminal effector memory RA)	CD3+ CD4+ CD25+ CD127low CD45RA+ CD27-
Act Treg (Activated)	CD3+ CD4+ CD25+ CD127low CD45RA- HLADR+
RTE Treg (Recent thymic emigrants)	CD3+ CD4+ CD25+ CD127low CD45RA+ CD31+
CD8	CD3+ CD8+
naïve CD8	CD3+ CD8+ CD45RA+ CD27+
CM CD8 (Central memory)	CD3+ CD8+ CD45RA- CD27+
EM CD8 (Effector memory)	CD3+ CD8+ CD45RA- CD27-
TemRA CD8 (Terminal effector memory RA)	CD3+ CD8+ CD45RA+ CD27-
Act CD8 (Activated)	CD3+ CD8+ CD45RA- HLADR+
RTE CD8 (Recent thymic emigrants)	CD3+ CD8+ CD45RA+ CD31+
Double positive CD4 D8 (DP)	CD4+ CD8+

**Table 4:** Cytokine levels before and after 6 weeks of anti-TNF therapy in primary responders and non-responders.

Cytokines	Week 0 (n = 74)			Week 6 (n = 69)		
	R (*n* = 66)	NR (*n* = 7)	*p* Value	R (*n* = 61)	NR (*n* = 7)	*p* Value
IFNγ, median (IQR, range)	3.65 (5.62; 0.37–21.90)	0.70 (4.91; 0.35–10.90)	0.10	1.63 (1.83; 0.15–10.30)	1.96 (3.08; 0.36–5.62)	0.77
IL-10, median (IQR, range)	3.36 (2.02; 1.47–34.10)	2.89 (4.59; 2.23–17.90)	0.79	3.51 (1.69; 1.54–86.00)	3.70 (1.60; 2.48–6.30)	0.33
IL-17A, median (IQR, range)	2.20 (1.92; 0–13.30)	3.03 (4.91; 1.02–13.90)	0.27	2.27 (1.40; 0.68–7.63)	2.31 (3.38; 1.10–40.40)	0.41
IL-4, median (IQR, range)	0.09 (0.09; 0–2.03)	0.08 (0.09; 0.01–0.50)	0.54	0.08 (0.08; 0.01–1.49)	0.09 (0.09; 0.03–0.52)	0.81

IQR, interquartile range; R, responders; NR, non-responders.

## Data Availability

Data will be uploaded and made accessible at “Repositorio de la Consejería de Sanidad de la Comunidad de Madrid”.

## References

[1] Guan Q. (2019). A Comprehensive Review and Update on the Pathogenesis of Inflammatory Bowel Disease. J. Immunol. Res..

[2] Khakoo N.S., Beecham A.H., Lyu J., Quintero M.A., Gomez L., Abreu M.T., Deshpande A.R., Kerman D.H., McCauley J.L., Proksell S. (2024). Early Life and Childhood Environmental Exposures, More Than Genetic Predisposition, Influence Age of Diagnosis in a Diverse Cohort of 2952 Patients with IBD. Clin. Gastroenterol. Hepatol..

[3] Hall C.H.T., de Zoeten E.F. (2024). Understanding very early onset inflammatory bowel disease (VEOIBD) in relation to inborn errors of immunity. Immunol. Rev..

[4] Gomez-Bris R., Saez A., Herrero-Fernandez B., Rius C., Sanchez-Martinez H., Gonzalez-Granado J.M. (2023). CD4 T-Cell Subsets and the Pathophysiology of Inflammatory Bowel Disease. Int. J. Mol. Sci..

[5] Lv J., Ibrahim Y.S., Yumashev A., Hjazi A., Faraz A., Alnajar M.J., Qasim M.T., Ghildiyal P., Hussein Zwamel A., Fakri Mustafa Y. (2024). A comprehensive immunobiology review of IBD: With a specific glance to Th22 lymphocytes development, biology, function, and role in IBD. Int. Immunopharmacol..

[6] Zhang S., Zhong R., Tang S., Chen L., Zhang H. (2024). Metabolic regulation of the Th17/Treg balance in inflammatory bowel disease. Pharmacol. Res..

[7] Jaeger N., Gamini R., Cella M., Schettini J.L., Bugatti M., Zhao S., Rosadini C.V., Esaulova E., Di Luccia B., Kinnett B. (2021). Single-cell analyses of Crohn’s disease tissues reveal intestinal intraepithelial T cells heterogeneity and altered subset distributions. Nat. Commun..

[8] Fujino S., Andoh A., Bamba S., Ogawa A., Hata K., Araki Y., Bamba T., Fujiyama Y. (2003). Increased expression of interleukin 17 in inflammatory bowel disease. Gut.

[9] Omenetti S., Pizarro T.T. (2015). The Treg/Th17 axis: A dynamic balance regulated by the gut microbiome. Front. Immunol..

[10] Li Z., Vermeire S., Bullens D., Ferrante M., Van Steen K., Noman M., Rutgeerts P., Ceuppens J.L., Van Assche G. (2015). Restoration of Foxp3 + Regulatory T-cell Subsets and Foxp3-Type 1 Regulatory-like T Cells in Inflammatory Bowel Diseases during Anti-tumor Necrosis Factor Therapy. Inflamm. Bowel Dis..

[11] Boschetti G., Nancey S., Sardi F., Roblin X., Flourié B., Kaiserlian D. (2011). Therapy with anti-TNFα antibody enhances number and function of Foxp3(+) regulatory T cells in inflammatory bowel diseases. Inflamm. Bowel Dis..

[12] Mitomi H., Ohkura Y., Yokoyama K., Sada M., Kobayashi K., Tanabe S., Fukui N., Kanazawa H., Kishimoto I., Saigenji K. (2007). Contribution of TIA-1+ and granzyme B+ cytotoxic T lymphocytes to cryptal apoptosis and ulceration in active inflammatory bowel disease. Pathol. Res. Pract..

[13] Neurath M.F. (2024). Strategies for targeting cytokines in inflammatory bowel disease. Nat. Rev. Immunol..

[14] Schett G., McInnes I.B., Neurath M.F. (2021). Reframing Immune-Mediated Inflammatory Diseases through Signature Cytokine Hubs. N. Engl. J. Med..

[15] El-Matary W., Carroll M.W., Deslandres C., Griffiths A.M., Kuenzig M.E., Mack D.R., Wine E., Weinstein J., Geist R., Davis T. (2023). The 2023 Impact of Inflammatory Bowel Disease in Canada: Special Populations-Children and Adolescents with IBD. J. Can. Assoc. Gastroenterol..

[16] Dulic S., Toldi G., Sava F., Kovács L., Molnár T., Milassin Á., Farkas K., Rutka M., Balog A. (2020). Specific T-Cell Subsets Can Predict the Efficacy of Anti-TNF Treatment in Inflammatory Bowel Diseases. Arch. Immunol. Ther. Exp..

[17] van Deventer S.J. (1999). Review article: Targeting TNF alpha as a key cytokine in the inflammatory processes of Crohn’s disease--the mechanisms of action of infliximab. Aliment. Pharmacol. Ther..

[18] Schnell A., Aicher C., Schnegelsberg P.A., Schwarz B., Schmidt H., Allabauer I., Rückel A., Regensburger A.P., Woelfle J., Hoerning A. (2024). Exhausted Lag-3+ CD4+ T cells are increased in pediatric Inflammatory Bowel Disease. Clin. Exp. Immunol..

[19] Salvador-Martín S., Zapata-Cobo P., Velasco M., Palomino L.M., Clemente S., Segarra O., Sánchez C., Tolín M., Moreno-Álvarez A., Fernández-Lorenzo A. (2023). Association between HLA DNA Variants and Long-Term Response to Anti-TNF Drugs in a Spanish Pediatric Inflammatory Bowel Disease Cohort. Int. J. Mol. Sci..

[20] Bosè F., Raeli L., Garutti C., Frigerio E., Cozzi A., Crimi M., Caprioli F., Scavelli R., Altomare G., Geginat J. (2011). Dual role of anti-TNF therapy: Enhancement of TCR-mediated T cell activation in peripheral blood and inhibition of inflammation in target tissues. Clin. Immunol..

[21] Zhang Y., Maksimovic J., Huang B., De Souza D.P., Naselli G., Chen H., Zhang L., Weng K., Liang H., Xu Y. (2018). Cord Blood CD8(+) T Cells Have a Natural Propensity to Express IL-4 in a Fatty Acid Metabolism and Caspase Activation-Dependent Manner. Front. Immunol..

[22] Sznurkowska K., Luty J., Bryl E., Witkowski J.M., Hermann-Okoniewska B., Landowski P., Kosek M., Szlagatys-Sidorkiewicz A. (2020). Enhancement of Circulating and Intestinal T Regulatory Cells and Their Expression of Helios and Neuropilin-1 in Children with Inflammatory Bowel Disease. J. Inflamm. Res..

[23] Shan J., Shi R., Hazra R., Hu X. (2024). Regulatory T lymphocytes in traumatic brain injury. Neurochem. Int..

[24] Ito M., Komai K., Mise-Omata S., Iizuka-Koga M., Noguchi Y., Kondo T., Sakai R., Matsuo K., Nakayama T., Yoshie O. (2019). Brain regulatory T cells suppress astrogliosis and potentiate neurological recovery. Nature.

[25] Nayer B., Tan J.L., Alshoubaki Y.K., Lu Y.-Z., Legrand J.M.D., Lau S., Hu N., Park A.J., Wang X.-N., Amann-Zalcenstein D. (2024). Local administration of regulatory T cells promotes tissue healing. Nat. Commun..

[26] Loffredo L.F., Savage T.M., Ringham O.R., Arpaia N. (2024). Treg-tissue cell interactions in repair and regeneration. J. Exp. Med..

[27] Vignali D.A.A., Collison L.W., Workman C.J. (2008). How regulatory T cells work. Nat. Rev. Immunol..

[28] Ueno A., Jijon H., Chan R., Ford K., Hirota C., Kaplan G.G., Beck P.L., Iacucci M., Fort Gasia M., Barkema H.W. (2013). Increased prevalence of circulating novel IL-17 secreting Foxp3 expressing CD4+ T cells and defective suppressive function of circulating Foxp3+ regulatory cells support plasticity between Th17 and regulatory T cells in inflammatory bowel disease patient. Inflamm. Bowel Dis..

[29] Jacobse J., Li J., Rings E.H.H.M., Samsom J.N., Goettel J.A. (2021). Intestinal Regulatory T Cells as Specialized Tissue-Restricted Immune Cells in Intestinal Immune Homeostasis and Disease. Front. Immunol..

[30] Duclaux-Loras R., Boschetti G., Flourie B., Roblin X., Leluduec J.-B., Paul S., Almeras T., Ruel K., Buisson A., Bienvenu J. (2022). Relationships of circulating CD4+ T cell subsets and cytokines with the risk of relapse in patients with Crohn’s disease. Front. Immunol..

[31] Kryczek I., Wu K., Zhao E., Wei S., Vatan L., Szeliga W., Huang E., Greenson J., Chang A., Roliński J. (2011). IL-17+ regulatory T cells in the microenvironments of chronic inflammation and cancer. J. Immunol..

[32] Laukova M., Glatman Zaretsky A. (2023). Regulatory T cells as a therapeutic approach for inflammatory bowel disease. Eur. J. Immunol..

[33] Raphael I., Nalawade S., Eagar T.N., Forsthuber T.G. (2014). T cell subsets and their signature cytokines in autoimmune and inflammatory diseases. Cytokine.

[34] Dahlén R., Strid H., Lundgren A., Isaksson S., Raghavan S., Magnusson M.K., Simrén M., Sjövall H., Öhman L. (2013). Infliximab inhibits activation and effector functions of peripheral blood T cells in vitro from patients with clinically active ulcerative colitis. Scand. J. Immunol..

[35] Verstockt S., Ver Donck F., Verstockt B., Glorieus E., De Decker M., Ballet V., Van Assche G., Laukens D., Ferrante M., Mana F. (2019). Up-regulation of IL17-related pathways in affected colon from ulcerative colitis compared with Crohn’s disease. J. Crohn’s Colitis.

[36] Menesy A., Hammad M., Aref S., Abozeid F.A.M. (2024). Level of interleukin 17 in inflammatory bowel disease and its relation with disease activity. BMC Gastroenterol..

[37] Kobayashi T., Okamoto S., Hisamatsu T., Kamada N., Chinen H., Saito R., Kitazume M.T., Nakazawa A., Sugita A., Koganei K. (2008). IL23 differentially regulates the Th1/Th17 balance in ulcerative colitis and Crohn’s disease. Gut.

[38] Cui G., Fan Q., Li Z., Goll R., Florholmen J. (2021). Evaluation of anti-TNF therapeutic response in patients with inflammatory bowel disease: Current and novel biomarkers. EBioMedicine.

[39] Hovhannisyan Z., Treatman J., Littman D.R., Mayer L. (2011). Characterization of interleukin-17-producing regulatory T cells in inflamed intestinal mucosa from patients with inflammatory bowel diseases. Gastroenterology.

[40] Sandy N.S., Marega L.F., Bechara G.D., Riccetto A.G.L., Bonfim C., Vilela M.M.D.S., Ribeiro A.F., Servidoni M.D.F., Lomazi E.A. (2021). Elevated IgA and IL-10 levels in very-early-onset inflammatory bowel disease secondary to IL-10 receptor deficiency. Rev. Paul. Pediatr..

[41] Girardelli M., Basaldella F., Paolera S.D., Vuch J., Tommasini A., Martelossi S., Crovella S., Bianco A.M. (2018). Genetic profile of patients with early onset inflammatory bowel disease. Gene.

[42] El-Hussuna A., Varghese C., Bhat V., Qvist N. (2022). Inflammatory Response in Patients with Crohn’s Disease Compared with Ulcerative Colitis: Secondary Results of a Prospective Pilot Study. Crohn’s Colitis 360.

[43] Toptygina A.P., Semikina E.L., Bobyleva G.V., Miroshkina L.V., Petrichuk S.V. (2014). Cytokine profile in children with inflammatory bowel disease. Biochemistry.

[44] Jessen B., Rodriguez-Sillke Y., Sonnenberg E., Schumann M., Kruglov A., Freise I., Schmidt F., Maul J., Kühl A.A., Glauben R. (2021). Level of Tumor Necrosis Factor Production by Stimulated Blood Mononuclear Cells Can Be Used to Predict Response of Patients with Inflammatory Bowel Diseases to Infliximab. Clin. Gastroenterol. Hepatol..

[45] Salvador-Martín S., López-Cauce B., Nuñez O., Laserna-Mendieta E.J., García M.I., Lobato E., Abarca-Zabalía J., Sanjurjo-Saez M., Lucendo A.J., Marín-Jiménez I. (2019). Genetic predictors of long-term response and trough levels of infliximab in crohn’s disease. Pharmacol. Res..

